# Face-specific negative bias of aesthetic perception in depression: Behavioral and EEG evidence

**DOI:** 10.3389/fpsyt.2023.1102843

**Published:** 2023-02-06

**Authors:** Zhitang Chen, Zhenghua Wang, Yuhua Shen, Suhua Zeng, Xiangyu Yang, Yifang Kuang, Zheng Dou, Lihui Wang, Weidong Li

**Affiliations:** ^1^Bio-X Institutes, Key Laboratory for the Genetics of Development and Neuropsychiatric Disorders, School of Life Sciences and Biotechnology, Shanghai Jiao Tong University, Shanghai, China; ^2^Institute of Psychology and Behavioral Science, Shanghai Jiao Tong University, Shanghai, China; ^3^WLA Laboratories, World Laureates Association, Shanghai, China; ^4^The Fourth People’s Hospital of Wuhu, Wuhu, Jiangsu, China; ^5^Shanghai Key Laboratory of Psychotic Disorders, Shanghai Mental Health Center, Shanghai Jiao Tong University School of Medicine, Shanghai, China; ^6^Shanghai Center for Brain Science and Brain-Inspired Intelligence Technology, Shanghai, China

**Keywords:** depression, face, aesthetic bias, electroencephalography, neuroaesthetics

## Abstract

**Introduction:**

Symptoms of depression are associated with the dysfunction of neural systems such as the emotion, reward system, and the default mode network. These systems were suggested by the model of neuroaesthetics as key contributions to aesthetic experience, leading to the prediction of atypical aesthetic orientation in depression. Here we investigated the aesthetic characteristics of depression and the corresponding neural underpinnings.

**Methods:**

Fifty-two (25 depression patients, 27 healthy controls) participants were asked to make aesthetic judgments on faces and landscapes in an electroencephalographic (EEG) experiment.

**Results:**

Our results indicate that relative to the controls, the depression tended to give ugly judgments and refrained from giving beautiful judgments, which was observed only for faces but not for landscapes. We also found that the face-induced component N170 was more negative in the depression group than the control group for ugly and neutral faces. Moreover, the aesthetic evaluation of ugly faces was associated with decreased N200 negativity in the depression group than in the control group, while the evaluation of beautiful faces was associated with decreased brain synchronization at the theta band.

**Discussion:**

These results suggested a face-specific negative aesthetic bias in depression which can help to design and develop aesthetics-oriented schemes in assisting the clinical diagnosis and therapy of depression.

## 1. Introduction

When people have a depressive episode, the visual world seems to lose its color. What was beautiful may look flat or even ugly. What was enjoyed or assigned value stops being pleasurable or worth living for. In accordance with the two core clinical symptoms “depressed mood” and “loss of interest or pleasure in nearly all activities” (i.e., anhedonia) (1), great effort has been made to reveal the cognitive and neural mechanisms of the affective and the motivational abnormalities in depression. On the affective aspect, depressive populations exhibit biased cognitive processing for negative information against positive information (2). At the neural level, the processing of negative information in depression is accompanied by hyperactivity in the limbic areas such as the amygdala, and aberrant activity in the prefrontal cortex (3). On the motivational aspect, depressive populations show insensitivity to reward stimuli and impaired ability to pursue rewarding behaviors, underlined by hypoactivity in the striatum and the prefrontal cortex (4).

While the affective and motivational characteristics of depression have been well documented, little is known about the aesthetic characteristics of depression. The aesthetic experience, which covers both the affective and motivational components, is linked to both the emotional and reward circuits in the brain (i.e., the emotion-valuation system) (5). A growing body of studies has shown that the aesthetic perception of various stimuli consistently activated the orbitofrontal cortex (6–9), an area crucially involved in both emotion and reward processing (10). Of note, the dysfunction of OFC has been suggested as responsible for both the bias for negative emotion and the anhedonia in depression (11–13). Moreover, depression is also characterized by pathological self-referential processes (i.e., depressive rumination) related to the dysfunction of the default mode network (DMN) (14). In parallel, the DMN was active during aesthetic appreciation and was suggested to reflect the internal processing evoked by the appreciated stimuli (15, 16). Therefore, the aesthetic orientation and the corresponding neural underpinnings may provide an integrated signature of the multidimensional symptoms of depression.

In the present study, we asked if people with clinically diagnosed depression have aesthetic abnormalities that can be probed with both behavioral and electroencephalographic (EEG) techniques. For this purpose, in an EEG experiment, we asked both depression and the healthy controls to make aesthetic judgments on faces and landscapes, stimuli commonly used in recent neuroaesthetic studies (17). Based on the negative emotional bias and the anhedonia in depression, we predicted that the depression group would tend to give ‘non-beautiful’ judgments on stimuli that agreed to be beautiful by healthy people. At the neural level, it has been shown in previous studies that the aesthetic experience engaged both the perceptual activity and the prefrontal activity in the brain. For instance, viewing beautiful faces enhanced the activity in the fusiform face area (FFA) (18, 19) and the OFC (9). In terms of EEG evidence, the well-known face-specific component N170 (20) was modulated by the aesthetic properties such as facial attractiveness (21) and emotion (22). A frontal negativity was elicited by the stimuli only in the aesthetic task (aesthetic judgment), but not in the perceptual task (symmetry judgment) (23). We hence expected that the biased aesthetic tendency in depression would be underlined by the corresponding EEG activities over the occipital and frontal regions.

## 2. Method

### 2.1. Participants

Sample size was determined by the sample size in recent studies on neuroaesthetics (*N* = 21–24, (7, 15, 19)), the availability of the recruited participants and the inclusion–exclusion criteria. Twenty-five depressive patients and 27 healthy controls (Table 1) participated in the EEG experiment. Patients were recruited from the outpatient clinics of the Fourth People’s Hospital of Wuhu. Diagnosis was performed by licensed psychiatrists using structured interview following DSM-V (24). Patients were included if they met the criteria of depression according to the structured interview. Patients were excluded if they met the criteria of schizophrenia, schizoaffective disorder, bipolar disorder, or anxiety disorder as the primary diagnosis. Patients were also excluded if their age lied outside the range of 18–40 years old. The healthy controls were recruited *via* internet-based advertisement and was screened for current and the history of psychiatric and neurological disorders. All participants had normal or corrected-to-normal vision. In addition to the clinical interview, both groups filled out the 21-item Beck Depression Inventory (25) prior to the experiment. Informed consent was obtained from all participants prior to the experiment. This study was approved by the Institutional Review Board for Human Research Protections of Shanghai Jiao Tong University (B2020011I).

**Table 1:** Demographic and clinical characteristics of participants (mean ± SD).

	Control	Depression	Statistics
Gender (F/M)	14/13	14/11	*p* = 0.76
Age (years)	24 ± 5.27	25.48 ± 6.42	*p* = 0.37
BDI score	6.37 ± 5.32	24.32 ± 10.30	*p* < 0.001
Education (years)	15.63 ± 3.88	14.32 ± 1.65	*p* = 0.71
Medication(Y/N)	0/27	23/2	

BDI, Beck Depression Inventory-II (21-item).

### 2.2. Materials

Pictures were selected based on the aesthetic judgments from an independent group of healthy participants who did not take part in the EEG experiment. For the rating, thirty healthy participants (14 females, 25.93 ± 2.53 years old; 16 males 25.86 ± 3.11 years old) were recruited and made aesthetic judgement (beautiful, neutral vs. ugly) on a total of 267 pictures (72 landscape pictures, 99 male face pictures and 96 female pictures) collected from the internet. Prior to the aesthetic task, pictures were preprocessed such that they had the same visual resolution (72 pixels per inch), and the same size for each type (Landscape: 12° * 7.3° of visual angle, Face: 6° * 8° of visual angle). The background of each face was kept white. We used 50% as the threshold to decide the valence of the picture in the way that a picture was assigned to a specific valence category (e.g., beautiful) if more than 50% of the participants made the corresponding aesthetic judgment (e.g., more than 50% of participants gave the “beautiful” response). Within each valence category, the pictures were ranked based on the proportion of agreement, and 66 pictures were selected based on the rankings for the main experiment. For faces, there were 44 “beautiful” pictures, 44 “ugly” pictures, and 44 neutral pictures, with an equal amount (i.e., 22 pictures) of female and male faces under each of the three valence types. For landscape, there were 22 pictures under each valence type.

### 2.3. Experimental procedure

**Figure 1:**
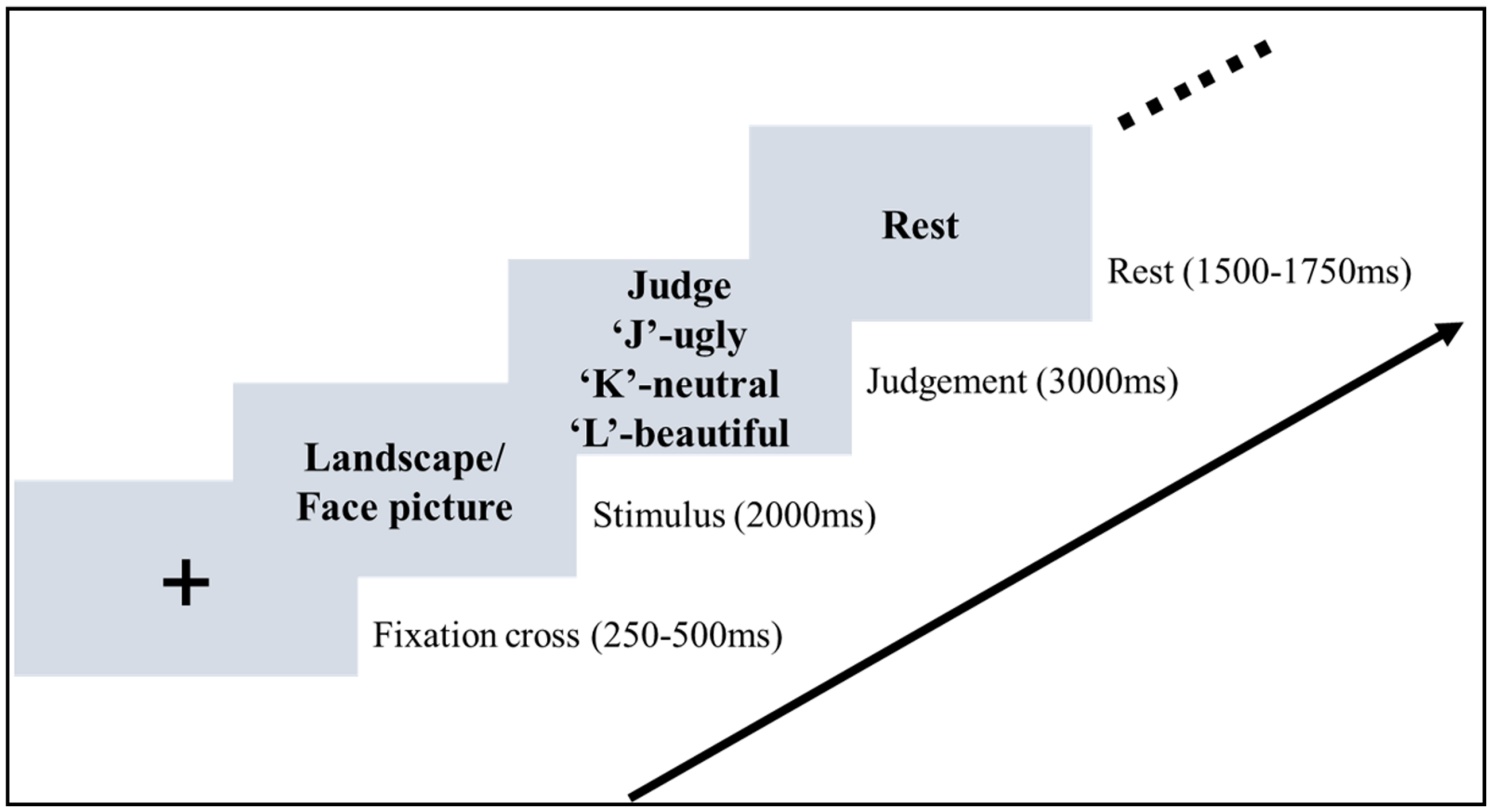
Stimuli sequence of the aesthetic judgment task. In the stimulus stage, participants were asked to pay attention to the picture and then evaluate the aesthetic valence in the judgement stage.

The formal experiment was conducted in a sound-attenuated room. Participants were seated in front of a monitor with an eye-to-monitor distance of 60 cm. Stimuli were presented at the center of a black background (Figure 1). At the beginning of each trial, a white cross was presented for a random interval of 0.25–0.5 s. A picture was then presented and remained on the screen for 2 s. Participants were asked to pay attention to the picture and evaluate the aesthetic valence of the picture. After the offset of the picture, the judgment frame was presented for 3 s. During the presentation of the judgment frame, the mapping between the three aesthetic valences and the response key was shown on the screen. Participants were required to press the key to make the aesthetic judgment (beautiful, neutral, vs. ugly). They were required to give a response within 3 s. The judgment frame was then replaced by the text “Rest” on the screen, indicating the end of the current trial. This rest frame was presented for a random interval of 1.5–1.75 s.

The 132 face pictures and 66 landscape pictures were mixed and pseudo-randomly assigned to 3 blocks of equal length, with each block including 22 male faces, 22 female faces, and 22 landscapes. Within each block, the 66 pictures were mixed and presented in random order. To ensure statistical power, each picture was presented twice and there were 6 blocks in total, with the first presentation in the first 3 blocks and the second presentation in the last 3 blocks. There was a break between every two blocks, which lasted 5–10 min. Participants were asked to avoid body movement and to reduce eye blinks during the task to minimize artifacts.

### 2.4. Statistical analysis of behavioral data

Per each participant and each experimental condition, the perceptual discriminability d’ and the response bias β and judgement criterion c were calculated based on the signal detection theory (26). Specifically, an aesthetic response was identified as Hit if it was consistent with the picture valence (e.g., an “ugly” response was given to an “ugly” picture), and a response was identified as False Alarm (FA) if it was inconsistent with the picture valence (e.g., an ugly response was given to a beautiful or a neutral picture). The perceptual discriminability d’ was then calculated using the formula d’ = Z(Hit rate) – Z(FA rate), and the response bias β was calculated using the formula β = exp. (d’ * c), where c = −(Z(Hit rate) + Z(FA rate))/2. A 2(Group: depression vs. control) * 2(Picture type: face vs. landscape) * 3(Picture valence: beautiful, ugly, vs. neutral) repeated-measures ANOVA was, respectively, conducted on d’, β and c, with Group included as the between-subjects factor and Picture type and Picture Valence as the within-subject factors.

Further separate ANOVAs and t tests were performed following an interaction that involved group. The Greenhouse–Geisser correction was used to compensate for sphericity violations and the Bonferroni method was used for correcting multiple comparisons. A threshold of α = 0.05 was used to decide statistical significance.

Reaction times (RTs) were calculated as the time of response relative to the onset of the judgement frame and were averaged across all trials for each experimental condition. The same 2 * 2 * 3 ANOVA was also performed on the mean RTs.

### 2.5. EEG recording and preprocessing

EEG signals were recorded with a NeuSen W332 system (Neuracle, China). Thirty-two Ag/AgCl scalp electrodes were placed according to the international 10/20 system. The impedance of each channel was kept below 5kΩ. The ground electrode was located at the FPz and the reference at the Cz. EEG signals were recorded with a sampling frequency of 1,000 Hz. A notch filter with 50 Hz was adopted to remove power frequency interference during data acquisition. Data were analyzed with EEG-toolbox (27). In the pre-processing stage, the offline raw data was amplified with a 0.5–100 Hz band-pass filter, down-sampled at 500 Hz/channel, and then re-referenced by the common average reference. Independent component analysis (ICA) algorithm was then used to remove the artifacts. Trials with peak-to-peak deflections exceeding ±100 μV were also excluded from data analysis.

### 2.6. Event-related potential analysis of EEG data

For each trial, data were segmented from −200 to 1,000 ms relative to the picture onset. Baseline corrections were applied to the interval of −200 to 0 ms relative to the picture onset. We focused on the occipital region over the visual cortex to examine if the aesthetic judgment was reflected by the perceptual processing, and the frontal region to assess how the aesthetic judgment was reflected by high-level cognitive processing. For this purpose, the channels (PO3, PO4, O1, Oz, O2) over the occipital region were grouped into a cluster, and the channels (FP1, FP2, F3, Fz, F4) over the frontal region were grouped into a cluster. For each cluster, the ERP times courses were averaged across all channels within the current cluster and averaged across all trials in a specific condition.

Given that face pictures and landscape pictures may induce distinct ERP components (e.g., face-specific N170), the statistical analysis was performed separately for faces and landscapes. The time range of each component was decided based on the peak amplitude. Specifically, the peak amplitude of each component was identified at the group level in each of the 6 conditions. For each component in each condition, the mean amplitude was calculated by averaging 100 ms centered at the peak point ((28); see Supplementary Table S1 for the time point of peak amplitude). For each picture type (Face and Landscape) and each cluster, the mean amplitude was entered into a 2(Group: depression vs. control) * 3(Picture valence: beautiful, ugly, vs. neutral) ANOVA. Only the occipital N170 and the frontal N200 were involved in the negative aesthetic bias (see results). The full ERP results are reported in Supplementary material.

For each of the two regions, the latencies of the first ERP component (Supplementary Table S2) were identified for each condition using the jackknife method (29). Considering the potential temporal correlation of the EEG waves, we performed the statistical analysis only on the latency of the first component. Latencies were also submitted to 2(Group: depression vs. control) * 3(Picture valence: beautiful, ugly, vs. neutral) ANOVA.

### 2.7. Time-frequency analysis of EEG data

For each trial, data were first subjected to a Butterworth bandpass filter (3rd order, 0.5–45 Hz), and then segmented from −500 to 2000 ms relative to the picture onset. The short-time Fourier transform (STFT, frequency range: 0.5–45 Hz, step = 1 Hz) with a Gaussian-tapered window (500 ms) was used to calculate the event-related spectral perturbations (ERSPs). The mean power in a baseline period (−500 to 0 ms relative to picture onset) was subtracted from each spectral estimate to produce the baseline-corrected ERSPs. The statistical testing was also focused on the occipital and frontal regions. To obtain ERSPs that related to the aesthetic evaluation, data were collapsed over all conditions for the occipital and the frontal region. Here we collapsed data over Face and Landscape conditions because there was no prior hypothesis for face-specific brain oscillations. To achieve unbiased statistical analysis, we firstly identified the oscillatory activities that involved the picture evaluation across groups, picture types, and valence types. For each region, a one-sample t-test was conducted for each cell of the ERSPs matrix, and cluster-based permutation testing (1,000 permutations, alpha level = 0.05) was used to correct multiple comparisons (30). Then, power amplitudes in each condition were extracted from the time-frequency clusters (see results for the significant clusters) that reached significance and were submitted to a 2(Group: depression vs. control) * 3(Picture valence: beautiful, ugly, vs. neutral) ANOVA for Face and Landscape, respectively. Only the frontal theta was involved in the negative aesthetic bias (see results). The full time-frequency results are reported in Supplementary material.

## 3. Results

### 3.1. Behavioral results

**Figure 2:**
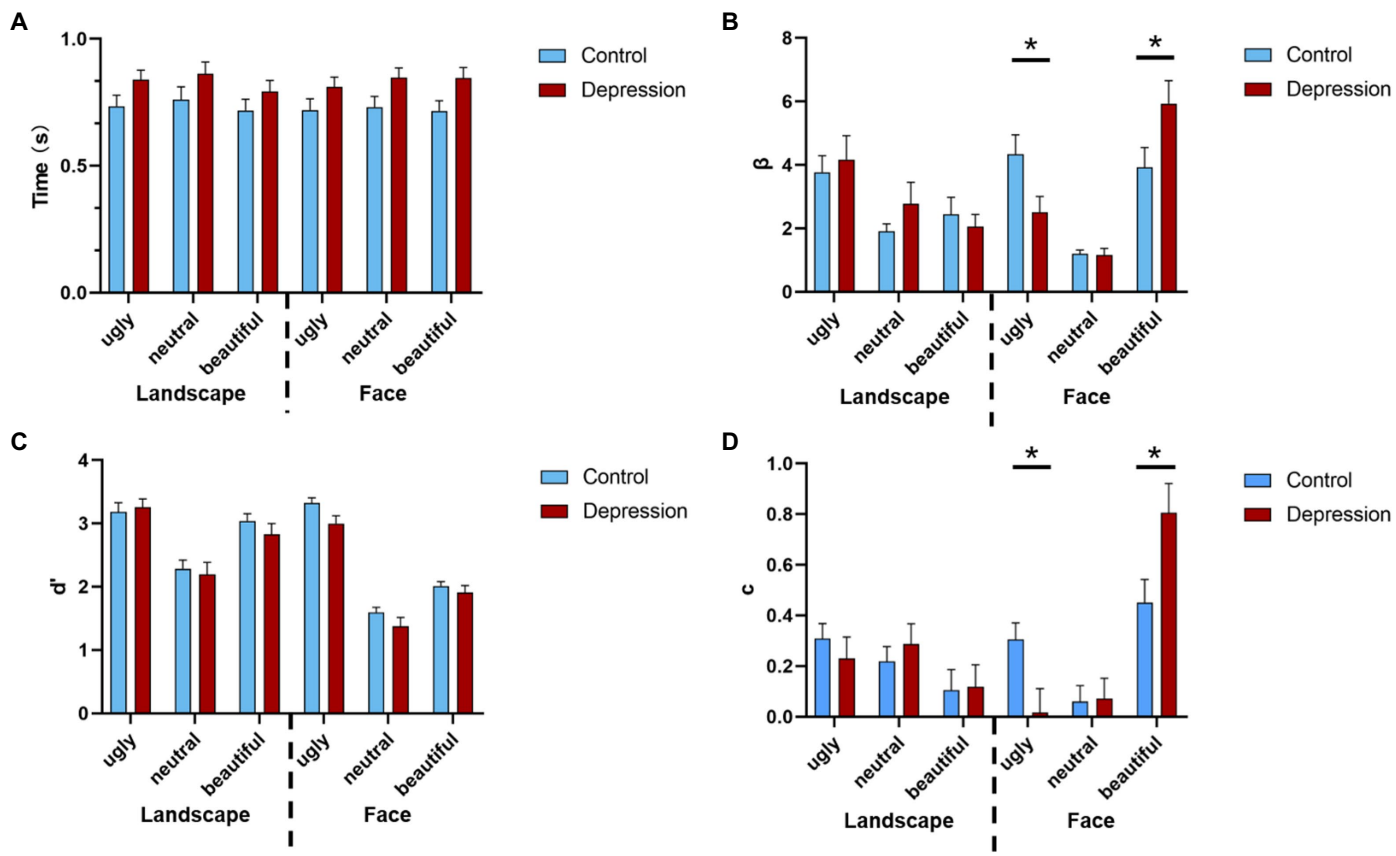
**(A)** The reaction times (RT) of the aesthetic judgments are shown as a function of the stimuli type, valence, and group. Error bars indicate standard errors. **(B)** The response bias is shown as a function of stimuli type, valence, and group. **(C)** The perceptual sensitivity is shown as a function of stimuli type, valence, and group, and **(D)** the judgement criterion is shown as a function of stimuli type, valence, and group. Error bars indicate standard errors. * indicates a significant difference.

The 2(Group: depression vs. control) * 2(Picture type: face vs. landscape) * 3(Picture valence: beautiful, neutral vs. ugly) ANOVA on response bias (measured by β) showed a three-way interaction, *F*(2, 100) = 6.447, *p* = 0.002, *η*^2^*_p_* = 0.114 (see Supplementary material for full ANOVA results). For Face, a separate 2 * 3 ANOVA with group as the between-subjects factor and valence as the within-subject factor showed a main effect of valence *F*(1.448, 72.384) = 21.007, *p* < 0.001, *η*^2^*_p_* = 0.296, with increased response bias from neutral to ugly, and from ugly to beautiful faces, all *p* < 0.033 (Bonferroni-corrected). Importantly, there was an interaction between group and valence, *F*(1.448, 72.384) = 5.435, *p* = 0.013, *η*^2^*_p_* = 0.098. This interaction was due to that the response bias for ugly faces was stronger in depression than the controls, *t*(50) = 2.286, *p* = 0.027, Cohen’s *d* = 0.635, 95% CI = [0.221, 3.425], whereas response bias for beautiful faces was weaker in depression than the controls, *t*(50) = 2.100, *p* = 0.041, Cohen’s *d* = 0.583, 95% CI = [0.087, 3.920] (Figure 2B). However, the decision bias for neutral faces did not differ between the two groups, *t* < 1. For Landscape, neither the main effect of group nor the interaction between group and valence reached significance, both *F* < 1. The same pattern was observed in both genders (Supplementary Figure S1). In all, the depressive group showed a stronger reaction bias towards ugly face.

The ANOVA on perceptual sensitivity (measured by d’) showed higher perceptual discriminability for ugly pictures than for beautiful pictures, and higher perceptual discriminability for beautiful pictures than for neutral pictures, *F*(2, 100) = 319.004, *p* < 0.001, *η*^2^*_p_* = 0.864, and *p* < 0.001 with Bonferroni-corrected comparisons (Figure 2C). The perceptual discriminability was higher for Landscape than for Face, *F*(1, 50) = 50.263, *p* < 0.001, *η*^2^*_p_* = 0.501. Also, the three-way interaction was significant, *F*(1.549, 77.459) = 3.408, *p* = 0.050, *η*^2^*_p_* = 0.064.

The ANOVA on judgement criterion (measured by c) showed a three-way interaction, *F*(2, 100) = 3.773, *p* = 0.026, *η*^2^*_p_* = 0.07. For Face, a separate 2 * 3 ANOVA showed a main effect of valence *F*(2, 100) = 16.789, *p* < 0.001, *η*^2^*_p_* = 0.251, with increased criterion from ugly to beautiful, and from neutral to beautiful faces, all *p* < 0.001 (Bonferroni-corrected). Also, there was an interaction between group and valence, *F*(2, 100) = 4.799, *p* = 0.010, *η*^2^*_p_* = 0.088, which was due to that the judgement criterion for ugly faces was weaker in depression than the controls, *t*(50) = 2.540, *p* = 0.014, Cohen’s *d* = 0.705, 95% CI = [0.061, 0.518], whereas criterion for beautiful faces was stronger in depression than the controls, *t*(50) = 2.424, *p* = 0.019, Cohen’s *d* = 0.673, 95% CI = [0.061, 0647]. However, the criterion for neutral faces did not differ between the two groups, *t* < 1. For Landscape, neither the main effect of group nor the interaction between group and valence reached significance, both *p* > 0.173. (Figure 2D). The ANOVA on RTs did not show any significant effect that involved Group, all *p* > 0.079 (Figure 2A). The hit and false alarm rate of beautiful, neutral, and ugly were shown in Table 2.

**Table 2:** The hit and false alarm rate under different respond of participants (mean ± SD).

		Landscape			Face		
		Ugly	Neutral	Beautiful	Ugly	Neutral	Beautiful
Hit rate	control	0.88 ± 0.12	0.79 ± 0.13	0.89 ± 0.11	0.89 ± 0.08	0.74 ± 0.13	0.68 ± 0.18
	depression	0.90 ± 0.08	0.74 ± 0.19	0.86 ± 0.19	0.90 ± 0.08	0.70 ± 0.19	0.54 ± 0.23
False alarm rate	control	0.04 ± 0.05	0.10 ± 0.08	0.07 ± 0.06	0.03 ± 0.04	0.21 ± 0.10	0.10 ± 0.07
	depression	0.05 ± 0.08	0.11 ± 0.12	0.08 ± 0.07	0.11 ± 0.15	0.24 ± 0.10	0.06 ± 0.06

### 3.2. ERP results

**Figure 3:**
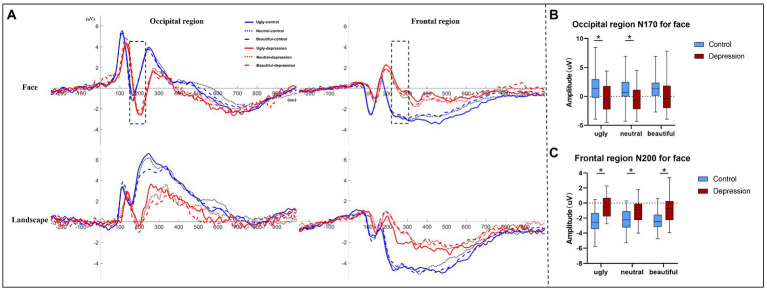
**(A)** Event-related potentials (ERP) averaged over the occipital region and the frontal region, respectively, are shown as a function of stimuli type, valence, and group. Only the two ERP components (occipital N170 and frontal N200) marked by dotted lines showed a significant interaction between group and valence. **(B)** The amplitudes of face-evoked occipital N170 are shown as a function of valence and group, and **(C)** the amplitudes of frontal N200 evoked by faces are shown as a function of valence and group. The error bars indicate the range of the amplitude values. The upper and lower boundaries of the colored square indicate the upper and lower quartiles of the amplitudes, and the black horizontal lines indicate the median of the amplitudes. * indicates a significant difference.

The depression group showed generally longer ERP latencies than the controls, all *p* < 0.001 (Figure 3A, Supplementary Table S2). For Face, the amplitude of the N170 component over the occipital region was more negative in depression than the controls, *F*(1, 50) = 6.313, *p* = 0.015, *η*^2^*_p_* = 0.112 (Figure 3B). There was also an interaction between group and valence, *F*(1.737, 86.863) = 3.925, *p* = 0.029, *η*^2^*_p_* = 0.073: the N170 was more negative in depression than the controls for ugly faces, *t*(50) = 2.787, *p* = 0.007, Cohen’s *d* = 0.774, 95% CI = [0.565, 3.479] and neutral faces, *t*(50) = 2.670, *p* = 0.010, Cohen’s *d* = 0.741, 95% CI = [0.450, 3.183], but not for beautiful faces, *t*(50) = 1.89, *p* = 0.065. In the depression group, there was a main effect of valence, *F*(2, 48) = 6.071, *p* = 0.004, *η*^2^*_p_* = 0.202, with more negative N170 to ugly faces (*p* = 0.087) and neutral faces (*p* = 0.016) than beautiful face, but no difference between ugly and neutral faces, *p* > 0.999 (Bonferroni-corrected). In the control group, however, the main effect of valence was not significant, *F*(2, 52) = 1.975, *p* = 0.149.

The amplitude of the N200 component over the frontal region showed an interaction between group and valence, *F*(2, 100) = 4.095, *p* = 0.020, *η*^2^*_p_* = 0.076 (Figure 3C). Further tests showed that the N200 was less negative in depression than the controls regardless of valence, all *p* < 0.004. In the depression group, there was a main effect of valence, *F*(2, 48) = 6.392, *p* = 0.003, *η*^2^*_p_* = 0.210, with less negative N200 to ugly faces than to neutral faces, *p* = 0.007, but no difference between ugly and beautiful faces, *p* = 0.132, or between neutral and beautiful faces, *p* = 0.512 (Bonferroni-corrected). By contrast in the control group, the main effect of valence did not reach significance, *F* < 1. Other ERP components did not show any interaction that involved Group for Face (Supplementary material).

No interaction that involved Group was observed for Landscape (Supplementary material).

### 3.3. EEG oscillation

**Figure 4:**
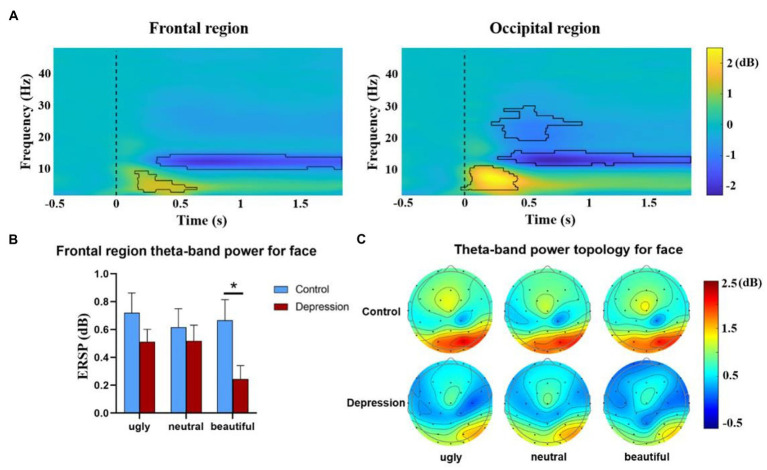
**(A)** The event-related spectral perturbations (ERSPs) over the frontal region (left panel) and the occipital region (right panel) were collapsed over stimuli type, valence and group, and are shown as a function of frequency and time. The areas marked by black lines indicate the time-frequency clusters that showed significant power changes relative to baseline. The 0-time point (marked by dotted lines) refers to the onset of the picture. **(B)** The ERSPs extracted from the frontal theta (the significant time-frequency range shown in **(A)** for faces are shown as a function of valence and group). Error bars indicate standard errors. *indicates a significant difference, and **(C)** the topographical distributions of the theta-power change extracted from the significant time-frequency range for faces (only for illustration).

The ERSPs over the frontal region and the occipital region were shown in Figure 4A, whereas only the frontal theta oscillation was involved in the aesthetic bias. For Face, the ANOVA on the frontal theta oscillation showed that the main effect of group was not significant, *F*(1, 50) = 2.318, *p* = 0.134. Both the main effect of valence, *F*(1.75, 87.514) = 3.283, *p* = 0.049, *η*^2^*_p_* = 0.062, and the interaction between valence and group, *F*(1.75, 87.514) = 3.277, p = 0.049, *η*^2^*_p_* = 0.062, were significant. The theta synchronization was weaker in the depression group than in the control group for beautiful faces, *t*(44.247) = 2.382, *p* = 0.022, Cohen’s *d* = 0.650, 95% CI = [0.065, 0.779], but not for ugly *t*(43.102) = 1.235, *p* = 0.224, or neutral faces, *t* < 1. In the depression group, there was a main effect of valence, *F*(2, 48) = 6.578, *p* = 0.003, *η*^2^*_p_* = 0.215, with lower synchronization to beautiful faces than ugly (*p* = 0.011) and neutral faces (*p* = 0.017), but no difference between neutral and ugly faces, *p* > 0.999 (Bonferroni-corrected) (Figure 4B). By contrast in the control group, the main effect of valence did not reach significance, *F* < 1. The topographical distributions of the theta-power change extracted from the significant time-frequency range for faces was shown in Figure 4C. The oscillatory activity at other bands did not show interaction that involved Group for Face (Supplementary material).

No interaction that involved Group was observed for Landscape (Supplementary material).

## 4. Discussion

In the present study, we investigated the aesthetic preference and the corresponding neural underpinnings in depression. The behavioral results showed a response bias and judgement criterion for faces but not for landscapes in depression. Specifically, relative to the control group, the depression group tended to give “ugly” judgments and were more conservative in giving “beautiful” judgments. The bias for negative judgment (i.e., ugly) while against positive judgment (i.e., beautiful) in depression was accompanied by enhanced N170 over the occipital cortex, decreased N200, and theta oscillation over the prefrontal cortex. These results convergingly point to a face-specific negative bias of aesthetic perception in depression.

As the response bias and judgment criteria reflect the strictness of the criteria used by the participants to judge the stimulus, that is, the greater β and c values represent the stricter judgment criteria. Compared with the control group, the depressed group showed significant abnormal criteria in judging the ugly and beautiful face, which meant that the depressed group had a negative bias specific to the face. Some studies on the response bias of depressed patients show that an overall increase in bias towards identifying emotions which appeared to be largely driven by results for mood-relevant emotions (31). The study on negative emotional bias in depression reports both increased attention and facilitated processing of negative stimuli, and reduced discrimination and withdrawal or avoidance from them (32). Although Lynn et al. reported that perceivers with poor sensitivity (d’) exhibited more extreme bias (c) than did perceivers with better sensitivity (33), we did not see a significant effect that involved group in sensitivity in our results, which may be related to methodology and the type and context of stimulus presentation. In addition, we did not find any significant effect related to group in RTs. The overall response time of the depression group was higher than that of the healthy group, which was consistent with the results of Anderson et al. (31), indicating that the impact of task difficulty could be ignored.

The negative aesthetic bias that specific to face in depression shown here raised the social origin of depression. While the cognitive model of depression highlighted the cognitive bias such as biased attention for negative information not exclusively social, the social-oriented model emphasized the interpersonal dysfunction such as interpersonal vulnerability and social difficulty (34). A recent study showed that face sensitivity was predicted by social cognition capabilities in healthy populations whereas was predicted by perceptual capabilities in major depressive disorder (35). Thus, the interpersonal dysfunctions in depression can sensitize the processing or reaction to social stimuli (e.g., faces) than non-social stimuli (e.g., landscapes), leading to a more pronounced bias.

The occipital N170 has been shown as a sensitive perceptual component to faces, with more negative amplitude to emotional faces than to neutral faces (22), and more negative amplitude to faces with higher attractiveness (21, 36). The face-induced N170 was also found to be more negative in depression than the controls for both emotional and neutral faces, indicating generally enhanced perceptual processing of faces in depression (37). Consistent with this result, here the ERP results showed more negative N170 during face processing in depression than the controls. Importantly, the degree of the enhanced N170 in depression increased from beautiful faces to neutral and ugly faces, a pattern echoed well with the decision bias of the aesthetic judgment, suggesting that the aesthetic evaluation modulates the perceptual representations of the encountered stimuli (18, 19).

The frontal N200 has been suggested as reflecting the initial affective reaction or contagion regardless of the emotional valence of the perceived stimuli (38, 39). For instance, the frontal N200 induced by faces conveying positive or negative emotion showed less negative amplitude than the N200 induced by neutral faces (40). Moreover, the frontal N200 was not restrictively involved in the processing of or reaction to face emotion, but also in face attractiveness, showing less negative amplitude to attractive faces than unattractive faces (41). Collectively, the frontal N200 may reflect the affective engagement of face processing, with less negative amplitude signaling higher affective engagement. Here the frontal N200 was generally less negative in the depression group than in the control group in the presence of all face stimuli. This result, along with the overall more negative occipital N170, suggested higher affective engagement in face processing in the depression group than in the controls. Importantly, ugly faces induced the least negative amplitude in depression, suggesting a biased affective engagement in ugly faces than in neutral and beautiful faces.

An alternative account could have been that the frontal N200 simply signaled the cognitive engagement rather than affective engagement in the aesthetic evaluation, as frontal N200 was often observed in tasks that required cognitive control (42). For instance, the frontal N200 was more negative when the presented stimuli contained incompatible information than when there was no incompatible information (42). On the contrary to this pattern, the frontal N200 showed less negative amplitude during high (vs. low) empathetic reactions to faces (39, 40). The opposite patterns of frontal N200 may reflect the external versus the internal processing, with more negative amplitude when the external processing was more dominant (e.g., during cognitive control) whereas less negative when the internal processing was more dominant (e.g., during affective reaction). This notion was supported by the current results that, relative to the control group, the frontal N200 was generally less negative in the depression group, who had a biased internal self-referential processing over external processing (14). Relative to beautiful and neutral faces, ugly faces in our experiment may concur with the negative self-referential information in depression and hence evoked the least negative N200.

As the frontal N200, the frontal theta oscillation was also found to be involved in both cognitive control and affective processing. On the one hand, the frontal theta was suggested as the source of the frontal N200 in cognitive control, with stronger synchronization in coping with high (vs. low) demand of control (28, 43). This account could be ruled out here as the frontal theta showed a different pattern from N200. Firstly, an overall group difference regardless of aesthetic valence was observed for N200 but not for theta synchronization. Secondly, while the decreased frontal N200 in depression manifested during the aesthetic evaluation of ugly faces, the desynchronized frontal theta oscillation in depression manifested during the aesthetic evaluation of beautiful faces. On the other hand, the frontal theta has been repeatedly found to show strong synchronization to stimuli with high emotional intensity (44). There are also findings that the theta synchronization was specifically associated with positive emotion (45, 46). In a more relevant study, greater theta synchronization was observed for the preferred face than the non-preferred face (47), which could originate from the activity in OFC (48). Along this line, the lowered theta synchronization, together with the behavioral bias against the positive judgment in depression, might be related to the lack of interest or the loss of pleasure in beautiful faces that are otherwise rewarding for healthy people.

One might argue that the negative aesthetic bias for face in depression could be simply due to the biased recognition of facial expressions in depression. It has been shown that depressive populations tended to perceive neutral expressions as negative (49) and had difficulty correctly identifying positive expressions (50). It should be noted, however, that the aesthetic experience of a face cannot be equal to the emotion identification of the face stimulus. While a neutral stimulus can be identified as sad, the sadness or sorrow conveyed by the stimulus can nevertheless be perceived as beautiful at the same time (51). In other words, a face with negative expression does not necessarily induce negative aesthetic experiences.

According to the recent models of neuroaesthetics, the emotion-valuation system and the DMN that supports self-referential processing are key contributors to the aesthetic experience (5). It is well-established that these systems are dysfunctional in depression, with manifestations such as the bias for negative emotion, the insensitivity to reward, and the ruminative thinking, as well as aberrance in the corresponding neural circuits (4, 13). Such dysfunctions could in combination lead to abnormal aesthetic orientation in depression. Specifically, in the present study, the insensitivity to reward and the dominance of negative over positive emotion may bias the perceptual processing away from the beautiful faces and result in a high threshold for “beautiful” judgments. The negative self-image and the dominance of internal over external processing may bias the perceptual processing of ugly faces and result in a low threshold for “ugly” judgments.

Taken together, the face-specific negative bias of aesthetic perception observed here, along with the EEG signatures, may be an integrated consequence of the multidimensional dysfunctions in depression.

## Data availability statement

The original contributions presented in the study are included in the article/Supplementary material, further inquiries can be directed to the corresponding authors.

## Ethics statement

The studies involving human participants were reviewed and approved by Institutional Review Board for Human Research Protections of Shanghai Jiao Tong University (B2020011I). The patients/participants provided their written informed consent to participate in this study.

## Author contributions

WL conceived the project. WL and LW supervised the study. ZC and ZD constructed the paradigm with faces and landscapes and collected data. ZW, YS, and SZ recruited the hospital samples. XY and YK recruited the healthy samples. ZC and LW analyzed data and wrote the first draft. All authors contributed to the article and approved the submitted version.

## Funding

This study is supported by Shanghai Education Commission Research and Innovation Program (2019-01-07-00-02-E00037), a Ministry Key Project (GW0890006), Shanghai Municipal Commission of Science and Technology Program (21dz2210100), “111” Program of Higher Education Discipline Innovation, and Shanghai Jiao Tong University Scientific and Technological Innovation Funds (B18034). LW was supported by the National Natural Science Foundation of China (32271086), and the Shanghai Sailing Program (20YF1422100).

## Conflict of interest

The authors declare that the research was conducted in the absence of any commercial or financial relationships that could be construed as a potential conflict of interest.

## Publisher’s note

All claims expressed in this article are solely those of the authors and do not necessarily represent those of their affiliated organizations, or those of the publisher, the editors and the reviewers. Any product that may be evaluated in this article, or claim that may be made by its manufacturer, is not guaranteed or endorsed by the publisher.
