# Supplementary material for: Face-specific negative bias of aesthetic perception in depression: Behavioral and EEG evidence
Source: Front Psychiatry. 2023 Feb 6;14:1102843. doi: 10.3389/fpsyt.2023.1102843 (PMC9939764; doi:10.3389/fpsyt.2023.1102843)
Supplement: Supplementary file 1 [file Data_Sheet_1.docx]

Supplementary Material

# Supplementary Results

## ERP results

In the occipital region, three main ERP components were involved in face processing: P1, N170, and P2. The 2 (Group: depression vs. control) * 3 (Valence: beautiful, ugly, vs. neutral) ANOVA on P1 amplitude did not show any significant main effects or interaction, all p > 0.101 (Fig. S2, left upper panel). The results of N170 are reported in the main text. The ANOVA on P2 revealed only a main effect of Group, F (1,50) = 7.970, p = 0.007, *η*^2^*_p_* = 0.137, with more positive amplitude in the control group than in the depression group. Neither the main effect of valence nor the interaction between group and valence reached significance, both p > 0.275.

In the occipital region, there were also three main ERP components involved in Landscape processing: P1, N2, and P2. The ANOVA on P1 did not show any significant effects, all p > 0.074 (Fig. S2, left lower panel). The ANOVA on N2 showed a main effect of group, F(1, 50) = 4.938, p = 0.031, *η*^2^*_p_* = 0.090, with more negative activity in the depression group than in the control group. Neither the main effect of valence nor the interaction between group and valence reached significance, both p > 0.089. The ANOVA on P2 revealed a main effect of group reached significance, F(1, 50) = 7.522, p = 0.008, *η*^2^*_p_* = 0.131, with more positive amplitude in the control group than in the depression group. There was also a main effect of picture valence, F(1.664, 83.185) = 5.374, p = 0.010, *η*^2^*_p_* = 0.097, with more positive amplitude for ugly (p = 0.024) and neutral scenes (p = 0.009) than beautiful scenes, but no difference between ugly and neutral scenes, p > 0.999 (Bonferroni-corrected). However, the interaction between Group and Valence did not reach significance, F (2, 100) = 1.273, p > 0.05.

In the frontal region, three main ERP components were involved in both Face and Landscape processing: N1, P2 and N2. For face, the ANOVA on N1 showed a main effect of group, F(1, 50) = 4.257, p = 0.044, *η*^2^*_p_* = 0.078, indicating more negative amplitudes in the control group compared to the depression group (Fig. S2, right upper panel). The main effect of picture valence was also significant, F(1.65, 82.506) = 5.785, p = 0.007, *η*^2^*_p_* = 0.104, with more negative amplitude to ugly than neutral (p = 0.019) and beautiful faces (p = 0.029), while no difference between neutral and beautiful faces, p > 0.999. However, the interaction between group and valence, did not reach significance, F < 1. The ANOVA on P2 showed a main effect of group, F(1, 50) = 13.597, p = 0.001, *η*^2^*_p_* = 0.214, with more positive activity in the depression group than in the control group. There was also a main effect of picture valence, F(2,100) = 9.495, p < 0.001, *η*^2^*_p_* = 0.160, with more positive amplitude for ugly than neutral (p = 0.015) and beautiful faces (p < 0.001), but no difference between neutral and beautiful faces, p = 0.494 (Bonferroni-corrected). The interaction between group and valence did not reach significance, F(2, 100) = 1.783, p = 0.173. The results of N2 (N200) are reported in the main text.

For Landscape, the 2*3 ANOVA on N1 did not show any significant effects, all p > 0.350 (Fig. S2, right lower panel). The ANOVA on P2 showed a main effect of group, F(1,50) = 7.789, p = 0.007, *η*^2^*_p_* = 0.135, with more positive activity in the depression group than in the control group. The other effects did not reach significance, all p > 0.075. The ANOVA on N2 showed only a main effect of Group, F(1, 50) = 13.955, p < 0.001, *η*^2^*_p_* = 0.218, with more negative activity in the control group than in the depression group. No other significant effect was observed, all p > 0.064.

## EEG oscillation

The occipital region showed significant ERSPs at theta, alpha, and beta bands. For Face, the ANOVA on theta oscillation revealed only a significant main effect of group, F(1, 50) = 8.411, p = 0.006, *η*^2^*_p_* = 0.144, with stronger theta synchronization in the control group than in the depression group (Fig. S3, left upper panel). Neither the main effect of picture valence nor the interaction reached significance, both p > 0.088. The ANOVA on alpha oscillation revealed only a significant main effect of group, F(1, 50) = 11.799, p = 0.001, *η*^2^*_p_* = 0.191, with stronger alpha desynchronization in the control group than in the depression group. Neither the main effect of picture valence nor the interaction reached significance, both p > 0.245. The ANOVA on beta oscillation revealed only a significant main effect of group, F(1, 50) = 7.511, p = 0.008, *η*^2^*_p_* = 0.131, with stronger beta desynchronization in the control group than in the depression group. Neither the main effect of picture valence nor the interaction reached significance, both p > 0.332.

For Landscape, the ANOVA on theta oscillation showed a main effect of group, F(1, 50) = 16.482, p < 0.001, *η*^2^*_p_* = 0.248, with stronger theta synchronization in the control group than in the depression group (Fig. S3, left lower panel). There was also a main effect of picture valence, F(2, 100) = 3.684, p = 0.029, *η*^2^*_p_* = 0.069, which was due to stronger theta synchronization induced by ugly scenes than by beautiful scenes, p = 0.043, while no significant difference between ugly and neutral scenes, p = 0.089, or between neutral and beautiful scenes, p > 0.999 (Bonferroni-corrected). However, the interaction between group and valence did not reach significance, F < 1. The ANOVA on alpha oscillation did not show any significant effects, all p > 0.063. The ANOVA on beta oscillation did not show any significant effects, all p > 0.064.

The frontal region showed significant ERSP at the theta and alpha band. For Face, the results of theta oscillation are reported in the main text. The ANOVA on alpha oscillation did not show any significant effects, all p > 0.052 (Fig. S3, right upper panel).

For Landscape, the ANOVA on theta oscillation showed only a main effect of group, F(1, 50) = 4.35, p = 0.042, *η*^2^*_p_* = 0.080, with weaker theta synchronization in the depression group than in the control group (Fig. S3, right lower panel). Neither the main effect of valence, F(1.756, 87.823) = 2.313, p = 0.112, nor the interaction, F < 1, reached significance. The ANOVA on alpha oscillation did not show any significant effects, all p > 0.056.

Supplementary material is not typeset so please ensure that all information is clearly presented, the appropriate caption is included in the file and not in the manuscript, and that the style conforms to the rest of the article. To avoid discrepancies between the published article and the supplementary material, please do not add the title, author list, affiliations or correspondence in the supplementary files.

# Supplementary Figures and Tables

## Supplementary Figures


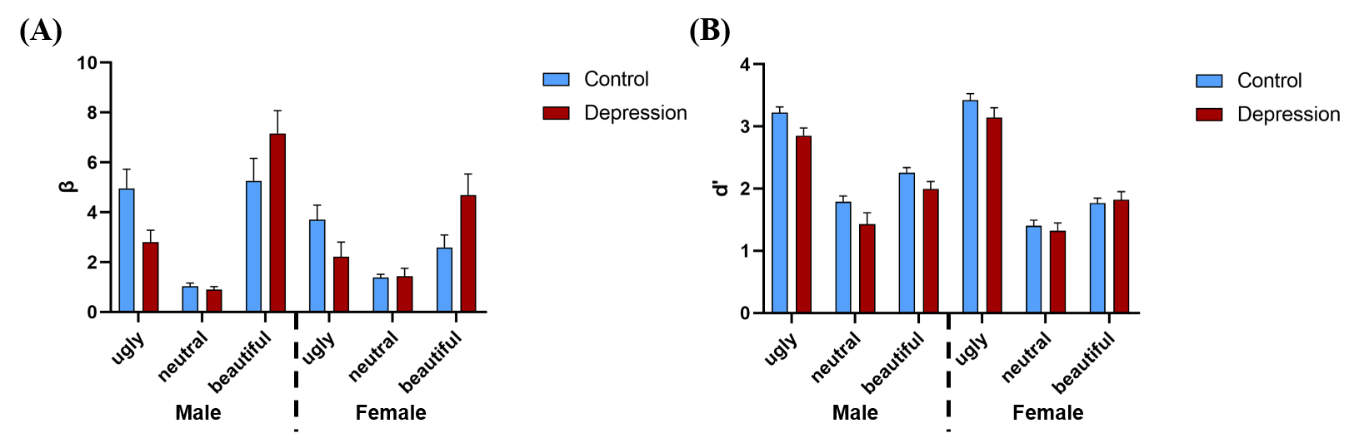


## Figure S1. (A) The response bias (β) and (B) the perceptual sensitivity (d’) and are shown as a function of the gender of the face stimuli and the gender of the two groups. Error bars indicate standard errors.

##
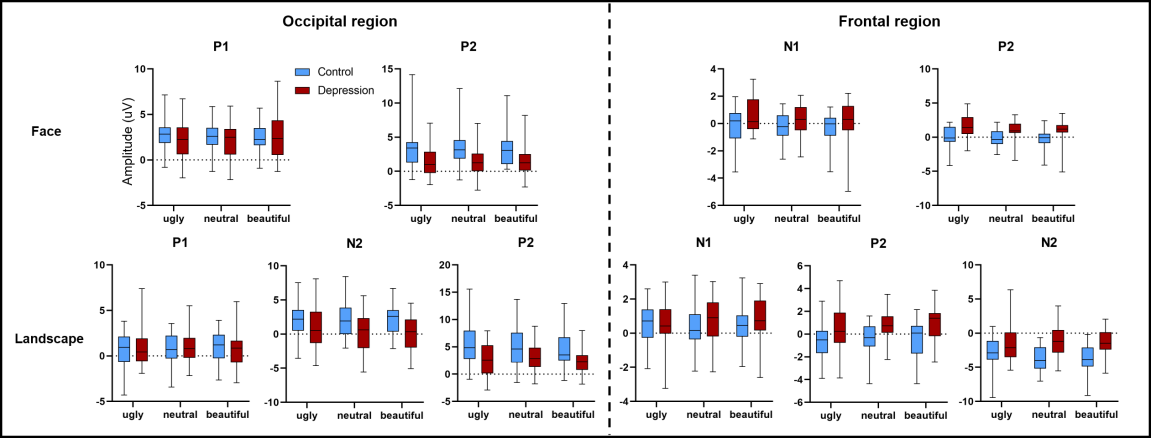


**Figure S2.** The amplitudes of ERPs over the occipital cortex (left) and the frontal cortex (right) that evoked by Face and Landscape are shown as a function of valence and group. The error bars indicate the range of the amplitude values. The upper and lower boundaries of the colored square indicate the upper and lower quartiles of the amplitudes, and the black horizontal lines indicate the median of the amplitudes.


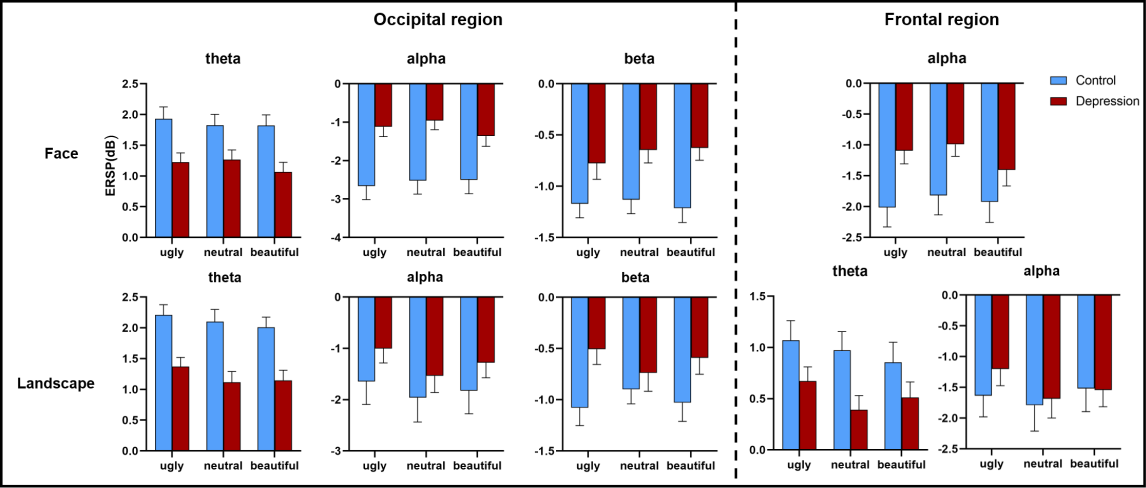


**Figure S3.** The event-related spectral perturbations (ERSPs) over the occipital region (left panel) and the frontal region (right panel) induced by Face and Landscape are shown as a function of valence and group. Error bars indicate standard errors.

## Supplementary Tables

**Table S1.** Peak time (ms, relative to picture onset) for each ERP component, condition and group.

|  |  |  | Occipital |  |  | Frontal |  |
| --- | --- | --- | --- | --- | --- | --- | --- |
|  |  | P1 | N170 | P2 | N1 | P2 | N2 |
|  | ugly | 110 | 166 | 248 | 114 | 166 | 260 |
| Face | neutral | 110 | 166 | 248 | 112 | 166 | 248 |
|  | beautiful | 110 | 162 | 246 | 114 | 166 | 260 |
| Control | |  |  |  |  |  |  |
|  | ugly | 112 | 154 | 244 | 114 | 160 | 228 |
| Landscape | neutral | 114 | 160 | 244 | 126 | 164 | 244 |
|  | beautiful | 116 | 158 | 250 | 122 | 162 | 248 |
|  | ugly | 132 | 196 | 272 | 134 | 192 | 276 |
| Face | neutral | 132 | 202 | 272 | 134 | 192 | 286 |
|  | beautiful | 130 | 192 | 274 | 136 | 190 | 274 |
| Depression | |  |  |  |  |  |  |
|  | ugly | 138 | 198 | 260 | 140 | 198 | 276 |
| Landscape | neutral | 140 | 188 | 284 | 148 | 190 | 274 |
|  | beautiful | 142 | 192 | 268 | 144 | 188 | 275 |

**Table S2.** The mean latencies (ms, mean ± SD) of P1 in the occipital region and of N1 in the frontal region.

|  |  | P1 | | N1 | |
| --- | --- | --- | --- | --- | --- |
|  |  | Control | Depression | Control | Depression |
| Face | ugly | 94.15 ± 12.56 | 114.80 ± 13.95 | 103.78 ± 5.56 | 123.12 ± 15.35 |
|  | neutral | 94.22 ± 12.73 | 112.96 ± 17.49 | 102.89 ± 5.09 | 123.68 ± 15.99 |
|  | beautiful | 94.44 ± 12.82 | 112.40 ± 18.06 | 103.41 ± 4.77 | 122.32 ± 16.20 |
| Landscape | ugly | 104.44 ± 18.43 | 123.92 ± 18.68 | 114.59 ± 16.46 | 129.44 ± 15.27 |
|  | neutral | 107.04 ± 21.08 | 120.00 ± 18.02 | 113.19 ± 14.96 | 134.48 ± 16.51 |
|  | beautiful | 101.70 ± 13.74 | 116.24 ± 19.72 | 112.44 ± 15.69 | 125.68 ± 18.12 |
